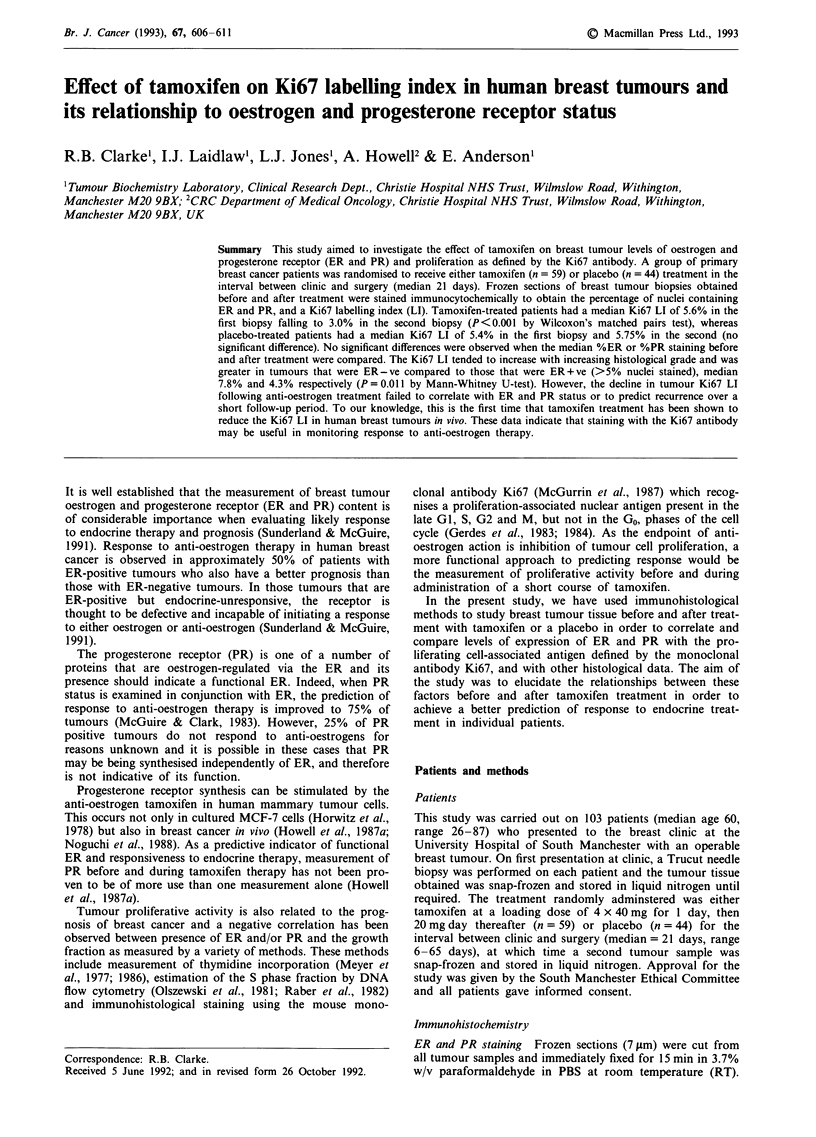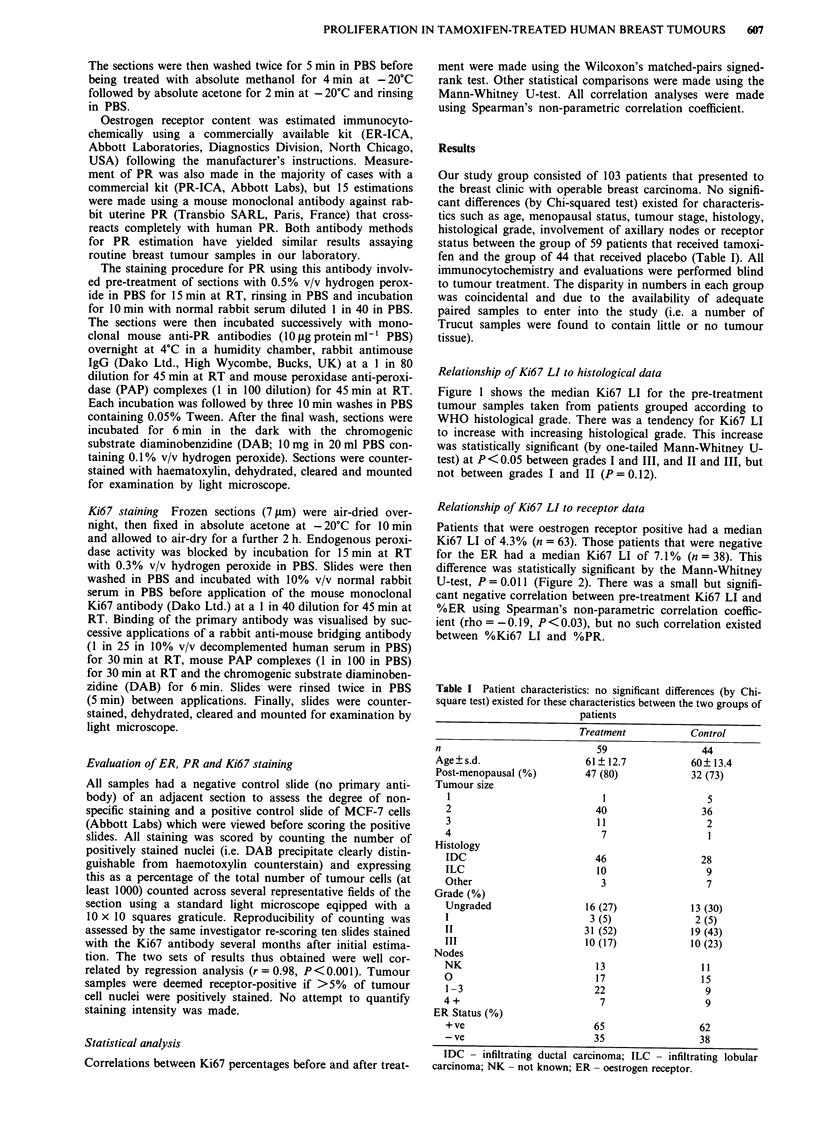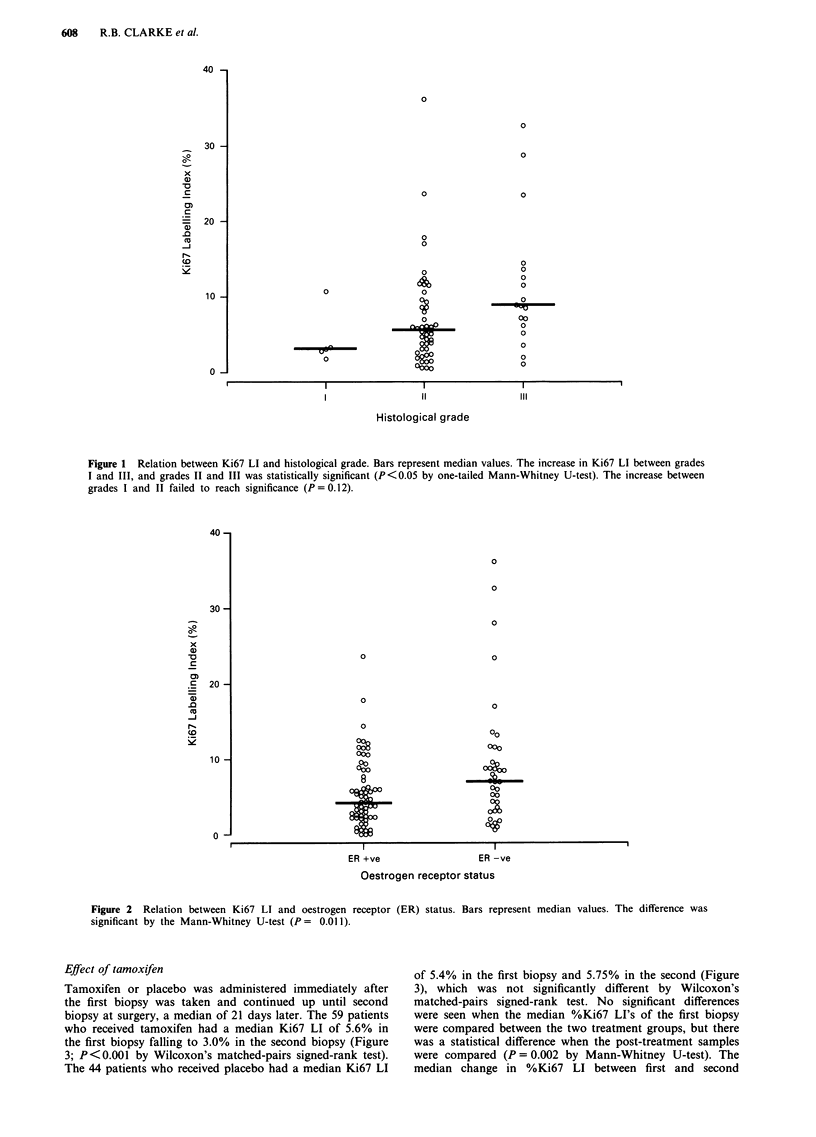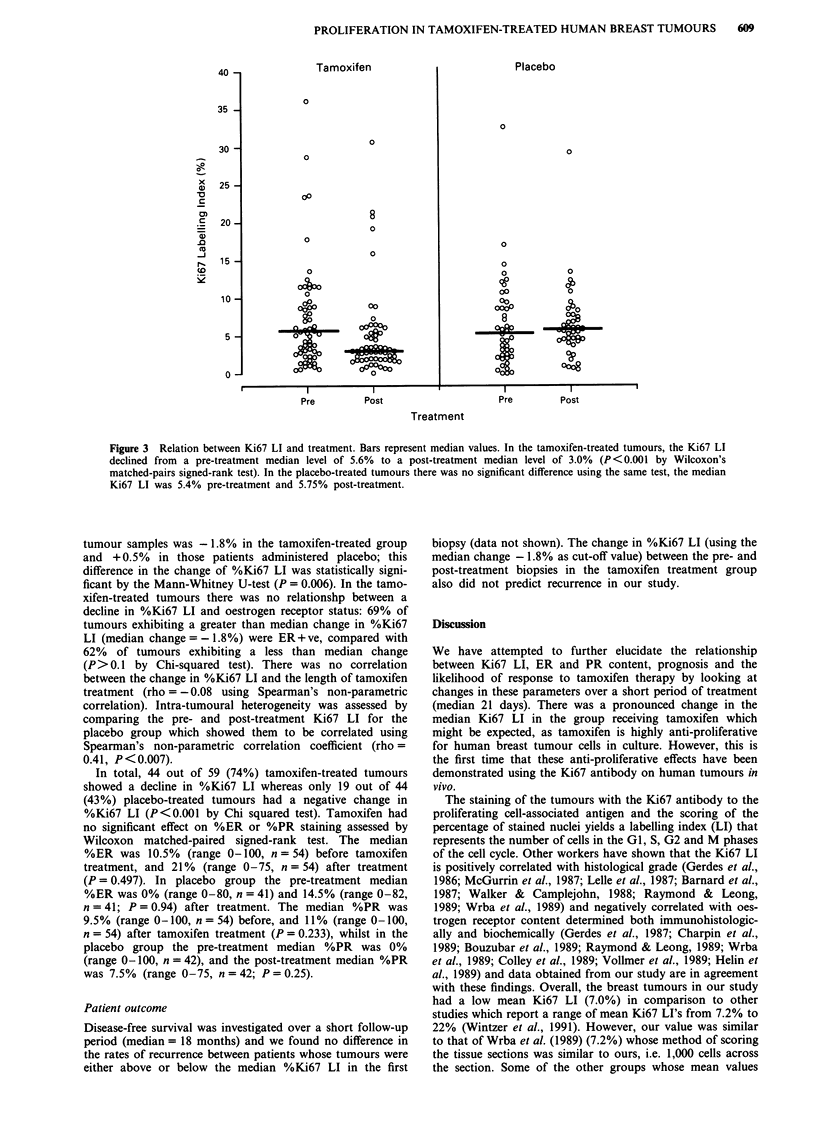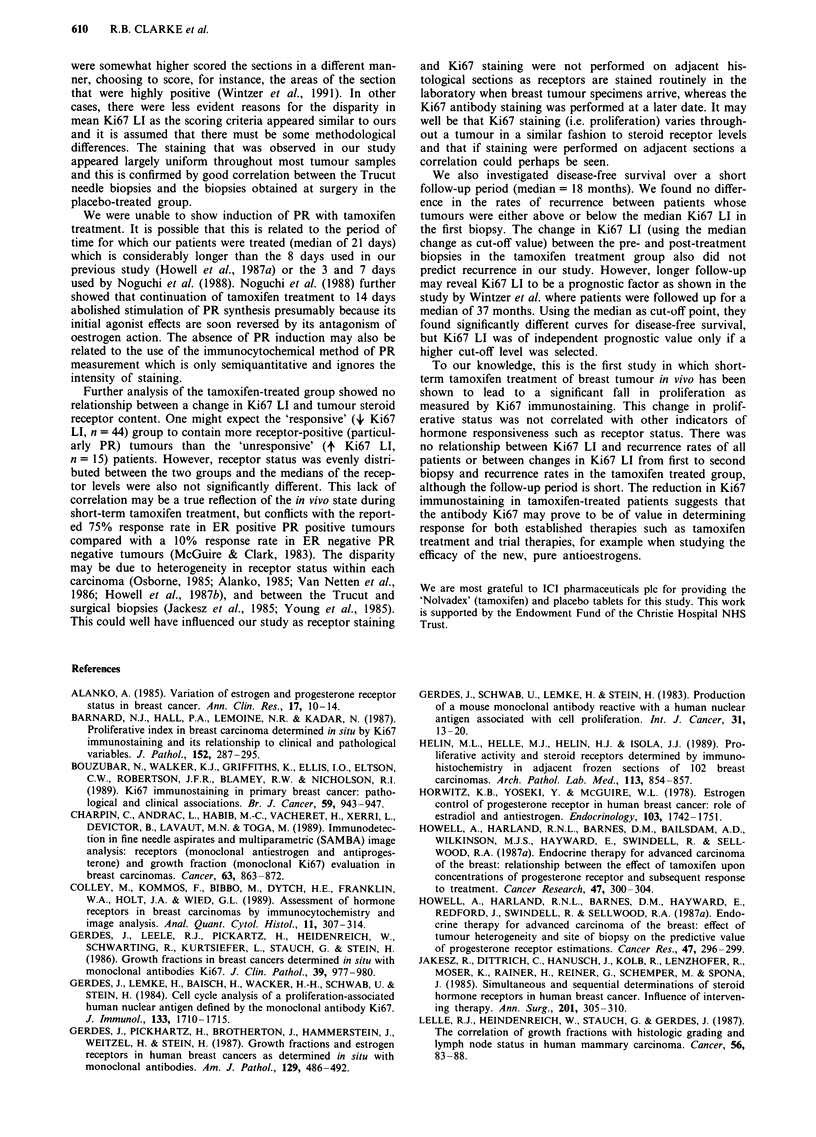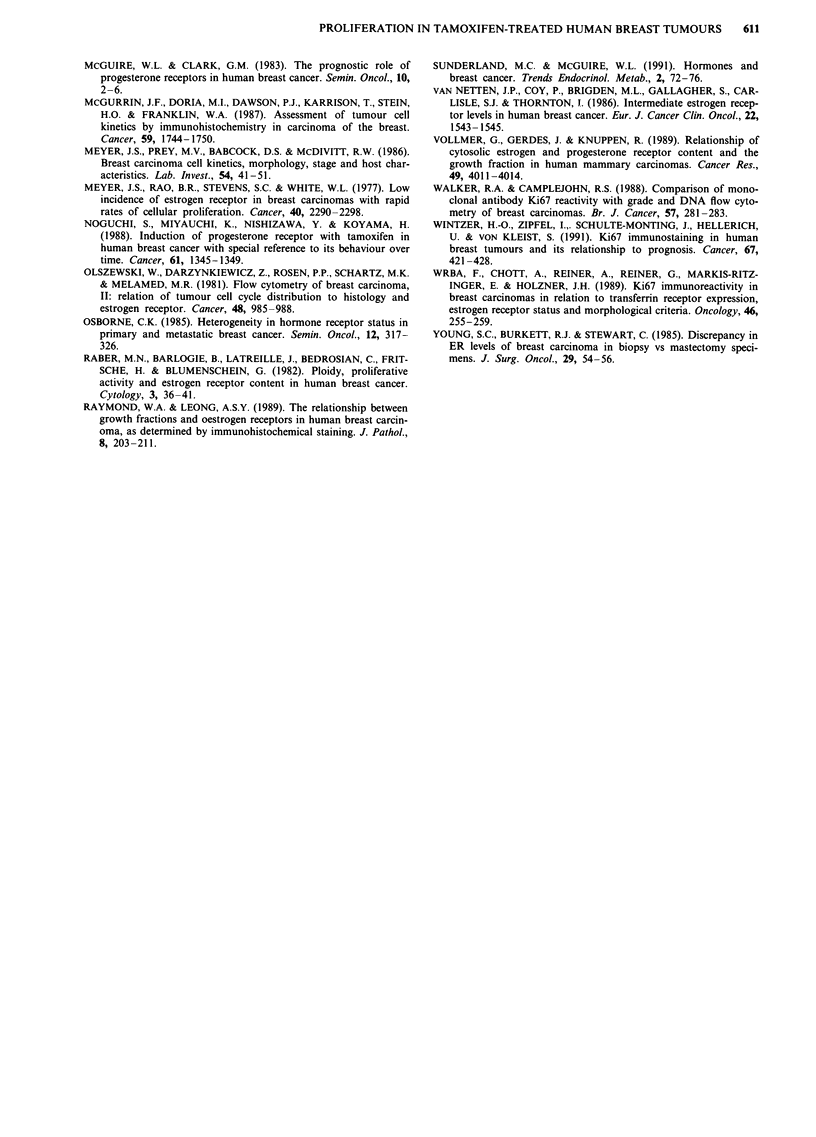# Effect of tamoxifen on Ki67 labelling index in human breast tumours and its relationship to oestrogen and progesterone receptor status.

**DOI:** 10.1038/bjc.1993.111

**Published:** 1993-03

**Authors:** R. B. Clarke, I. J. Laidlaw, L. J. Jones, A. Howell, E. Anderson

**Affiliations:** Clinical Research Dept., Christie Hospital NHS Trust, Withington, Manchester.

## Abstract

This study aimed to investigate the effect of tamoxifen on breast tumour levels of oestrogen and progesterone receptor (ER and PR) and proliferation as defined by the Ki67 antibody. A group of primary breast cancer patients was randomised to receive either tamoxifen (n = 59) or placebo (n = 44) treatment in the interval between clinic and surgery (median 21 days). Frozen sections of breast tumour biopsies obtained before and after treatment were stained immunocytochemically to obtain the percentage of nuclei containing ER and PR, and a Ki67 labelling index (LI). Tamoxifen-treated patients had a median Ki67 LI of 5.6% in the first biopsy falling to 3.0% in the second biopsy (P < 0.001 by Wilcoxon's matched pairs test), whereas placebo-treated patients had a median Ki67 LI of 5.4% in the first biopsy and 5.75% in the second (no significant difference). No significant differences were observed when the median %ER or %PR staining before and after treatment were compared. The Ki67 LI tended to increase with increasing histological grade and was greater in tumours that were ER - ve compared to those that were ER + ve (> 5% nuclei stained), median 7.8% and 4.3% respectively (P = 0.011 by Mann-Whitney U-test). However, the decline in tumour Ki67 LI following anti-oestrogen treatment failed to correlate with ER and PR status or to predict recurrence over a short follow-up period. To our knowledge, this is the first time that tamoxifen treatment has been shown to reduce the Ki67 LI in human breast tumours in vivo. These data indicate that staining with the Ki67 antibody may be useful in monitoring response to anti-oestrogen therapy.


Br. J. Cancer (1993), 67, 606–611 © Macmillan Press Ltd., 1993

### Effect of tamoxifen on Ki67 labelling index in human breast tumours and its relationship to oestrogen and progesterone receptor status

R.B. Clarke[1], I.J. Laidlaw[1], L.J. Jones[1], A. Howell[2] & E. Anderson[1]

[1]*Tumour Biochemistry Laboratory, Clinical Research Dept., Christie Hospital NHS Trust, Wilmslow Road, Withington, Manchester M20 9BX;* [2]*CRC Department of Medical Oncology, Christie Hospital NHS Trust, Wilmslow Road, Withington, Manchester M20 9BX, UK*

**Summary** This study aimed to investigate the effect of tamoxifen on breast tumour levels of oestrogen and progesterone receptor (ER and PR) and proliferation as defined by the Ki67 antibody. A group of primary breast cancer patients was randomised to receive either tamoxifen ($n = 59$) or placebo ($n = 44$) treatment in the interval between clinic and surgery (median 21 days). Frozen sections of breast tumour biopsies obtained before and after treatment were stained immunocytochemically to obtain the percentage of nuclei containing ER and PR, and a Ki67 labelling index (LI). Tamoxifen-treated patients had a median Ki67 LI of 5.6% in the first biopsy falling to 3.0% in the second biopsy ($P < 0.001$ by Wilcoxon's matched pairs test), whereas placebo-treated patients had a median Ki67 LI of 5.4% in the first biopsy and 5.75% in the second (no significant difference). No significant differences were observed when the median %ER or %PR staining before and after treatment were compared. The Ki67 LI tended to increase with increasing histological grade and was greater in tumours that were ER–ve compared to those that were ER+ve (>5% nuclei stained), median 7.8% and 4.3% respectively ($P = 0.011$ by Mann-Whitney U-test). However, the decline in tumour Ki67 LI following anti-oestrogen treatment failed to correlate with ER and PR status or to predict recurrence over a short follow-up period. To our knowledge, this is the first time that tamoxifen treatment has been shown to reduce the Ki67 LI in human breast tumours *in vivo*. These data indicate that staining with the Ki67 antibody may be useful in monitoring response to anti-oestrogen therapy.

It is well established that the measurement of breast tumour oestrogen and progesterone receptor (ER and PR) content is of considerable importance when evaluating likely response to endocrine therapy and prognosis (Sunderland & McGuire, 1991). Response to anti-oestrogen therapy in human breast cancer is observed in approximately 50% of patients with ER-positive tumours who also have a better prognosis than those with ER-negative tumours. In those tumours that are ER-positive but endocrine-unresponsive, the receptor is thought to be defective and incapable of initiating a response to either oestrogen or anti-oestrogen (Sunderland & McGuire, 1991).

The progesterone receptor (PR) is one of a number of proteins that are oestrogen-regulated via the ER and its presence should indicate a functional ER. Indeed, when PR status is examined in conjunction with ER, the prediction of response to anti-oestrogen therapy is improved to 75% of tumours (McGuire & Clark, 1983). However, 25% of PR positive tumours do not respond to anti-oestrogens for reasons unknown and it is possible in these cases that PR may be being synthesised independently of ER, and therefore is not indicative of its function.

Progesterone receptor synthesis can be stimulated by the anti-oestrogen tamoxifen in human mammary tumour cells. This occurs not only in cultured MCF-7 cells (Horwitz *et al.*, 1978) but also in breast cancer *in vivo* (Howell *et al.*, 1987*a*; Noguchi *et al.*, 1988). As a predictive indicator of functional ER and responsiveness to endocrine therapy, measurement of PR before and during tamoxifen therapy has not been proven to be of more use than one measurement alone (Howell *et al.*, 1987*a*).

Tumour proliferative activity is also related to the prognosis of breast cancer and a negative correlation has been observed between presence of ER and/or PR and the growth fraction as measured by a variety of methods. These methods include measurement of thymidine incorporation (Meyer *et al.*, 1977; 1986), estimation of the S phase fraction by DNA flow cytometry (Olszewski *et al.*, 1981; Raber *et al.*, 1982) and immunohistological staining using the mouse monoclonal antibody Ki67 (McGurrin *et al.*, 1987) which recognises a proliferation-associated nuclear antigen present in the late G1, S, G2 and M, but not in the $G_0$, phases of the cell cycle (Gerdes *et al.*, 1983; 1984). As the endpoint of anti-oestrogen action is inhibition of tumour cell proliferation, a more functional approach to predicting response would be the measurement of proliferative activity before and during administration of a short course of tamoxifen.

In the present study, we have used immunohistological methods to study breast tumour tissue before and after treatment with tamoxifen or a placebo in order to correlate and compare levels of expression of ER and PR with the proliferating cell-associated antigen defined by the monoclonal antibody Ki67, and with other histological data. The aim of the study was to elucidate the relationships between these factors before and after tamoxifen treatment in order to achieve a better prediction of response to endocrine treatment in individual patients.

### Patients and methods

### *Patients*

This study was carried out on 103 patients (median age 60, range 26–87) who presented to the breast clinic at the University Hospital of South Manchester with an operable breast tumour. On first presentation at clinic, a Trucut needle biopsy was performed on each patient and the tumour tissue obtained was snap-frozen and stored in liquid nitrogen until required. The treatment randomly adminstered was either tamoxifen at a loading dose of 4 × 40 mg for 1 day, then 20 mg day thereafter ($n = 59$) or placebo ($n = 44$) for the interval between clinic and surgery (median = 21 days, range 6–65 days), at which time a second tumour sample was snap-frozen and stored in liquid nitrogen. Approval for the study was given by the South Manchester Ethical Committee and all patients gave informed consent.

### *Immunohistochemistry*

*ER and PR staining* Frozen sections (7 µm) were cut from all tumour samples and immediately fixed for 15 min in 3.7% w/v paraformaldehyde in PBS at room temperature (RT).

Correspondence: R.B. Clarke.
Received 5 June 1992; and in revised form 26 October 1992.

The sections were then washed twice for 5 min in PBS before being treated with absolute methanol for 4 min at −20°C followed by absolute acetone for 2 min at −20°C and rinsing in PBS.

Oestrogen receptor content was estimated immunocytochemically using a commercially available kit (ER-ICA, Abbott Laboratories, Diagnostics Division, North Chicago, USA) following the manufacturer's instructions. Measurement of PR was also made in the majority of cases with a commercial kit (PR-ICA, Abbott Labs), but 15 estimations were made using a mouse monoclonal antibody against rabbit uterine PR (Transbio SARL, Paris, France) that cross-reacts completely with human PR. Both antibody methods for PR estimation have yielded similar results assaying routine breast tumour samples in our laboratory.

The staining procedure for PR using this antibody involved pre-treatment of sections with 0.5% v/v hydrogen peroxide in PBS for 15 min at RT, rinsing in PBS and incubation for 10 min with normal rabbit serum diluted 1 in 40 in PBS. The sections were then incubated successively with monoclonal mouse anti-PR antibodies (10 μg protein $ml^{-1}$ PBS) overnight at 4°C in a humidity chamber, rabbit antimouse IgG (Dako Ltd., High Wycombe, Bucks, UK) at a 1 in 80 dilution for 45 min at RT and mouse peroxidase anti-peroxidase (PAP) complexes (1 in 100 dilution) for 45 min at RT. Each incubation was followed by three 10 min washes in PBS containing 0.05% Tween. After the final wash, sections were incubated for 6 min in the dark with the chromogenic substrate diaminobenzidine (DAB; 10 mg in 20 ml PBS containing 0.1% v/v hydrogen peroxide). Sections were counterstained with haematoxylin, dehydrated, cleared and mounted for examination by light microscope.

*Ki67 staining* Frozen sections (7 μm) were air-dried overnight, then fixed in absolute acetone at −20°C for 10 min and allowed to air-dry for a further 2 h. Endogenous peroxidase activity was blocked by incubation for 15 min at RT with 0.3% v/v hydrogen peroxide in PBS. Slides were then washed in PBS and incubated with 10% v/v normal rabbit serum in PBS before application of the mouse monoclonal Ki67 antibody (Dako Ltd.) at a 1 in 40 dilution for 45 min at RT. Binding of the primary antibody was visualised by successive applications of a rabbit anti-mouse bridging antibody (1 in 25 in 10% v/v decomplemented human serum in PBS) for 30 min at RT, mouse PAP complexes (1 in 100 in PBS) for 30 min at RT and the chromogenic substrate diaminobenzidine (DAB) for 6 min. Slides were rinsed twice in PBS (5 min) between applications. Finally, slides were counterstained, dehydrated, cleared and mounted for examination by light microscope.

### *Evaluation of ER, PR and Ki67 staining*

All samples had a negative control slide (no primary antibody) of an adjacent section to assess the degree of non-specific staining and a positive control slide of MCF-7 cells (Abbott Labs) which were viewed before scoring the positive slides. All staining was scored by counting the number of positively stained nuclei (i.e. DAB precipitate clearly distinguishable from haemotoxylin counterstain) and expressing this as a percentage of the total number of tumour cells (at least 1000) counted across several representative fields of the section using a standard light microscope eqipped with a 10 × 10 squares graticule. Reproducibility of counting was assessed by the same investigator re-scoring ten slides stained with the Ki67 antibody several months after initial estimation. The two sets of results thus obtained were well correlated by regression analysis ($r = 0.98$, $P < 0.001$). Tumour samples were deemed receptor-positive if >5% of tumour cell nuclei were positively stained. No attempt to quantify staining intensity was made.

### *Statistical analysis*

Correlations between Ki67 percentages before and after treatment were made using the Wilcoxon's matched-pairs signed-rank test. Other statistical comparisons were made using the Mann-Whitney U-test. All correlation analyses were made using Spearman's non-parametric correlation coefficient.

### Results

Our study group consisted of 103 patients that presented to the breast clinic with operable breast carcinoma. No significant differences (by Chi-squared test) existed for characteristics such as age, menopausal status, tumour stage, histology, histological grade, involvement of axillary nodes or receptor status between the group of 59 patients that received tamoxifen and the group of 44 that received placebo (Table I). All immunocytochemistry and evaluations were performed blind to tumour treatment. The disparity in numbers in each group was coincidental and due to the availability of adequate paired samples to enter into the study (i.e. a number of Trucut samples were found to contain little or no tumour tissue).

### *Relationship of Ki67 LI to histological data*

Figure 1 shows the median Ki67 LI for the pre-treatment tumour samples taken from patients grouped according to WHO histological grade. There was a tendency for Ki67 LI to increase with increasing histological grade. This increase was statistically significant (by one-tailed Mann-Whitney U-test) at $P < 0.05$ between grades I and III, and II and III, but not between grades I and II ($P = 0.12$).

### *Relationship of Ki67 LI to receptor data*

Patients that were oestrogen receptor positive had a median Ki67 LI of 4.3% ($n = 63$). Those patients that were negative for the ER had a median Ki67 LI of 7.1% ($n = 38$). This difference was statistically significant by the Mann-Whitney U-test, $P = 0.011$ (Figure 2). There was a small but significant negative correlation between pre-treatment Ki67 LI and %ER using Spearman's non-parametric correlation coefficient (rho = −0.19, $P < 0.03$), but no such correlation existed between %Ki67 LI and %PR.

**Table I** Patient characteristics: no significant differences (by Chi-square test) existed for these characteristics between the two groups of patients

| | *Treatment* | *Control* |
|---|---|---|
| *n* | 59 | 44 |
| Age ± s.d. | 61 ± 12.7 | 60 ± 13.4 |
| Post-menopausal (%) | 47 (80) | 32 (73) |
| Tumour size | | |
| 1 | 1 | 5 |
| 2 | 40 | 36 |
| 3 | 11 | 2 |
| 4 | 7 | 1 |
| Histology | | |
| IDC | 46 | 28 |
| ILC | 10 | 9 |
| Other | 3 | 7 |
| Grade (%) | | |
| Ungraded | 16 (27) | 13 (30) |
| I | 3 (5) | 2 (5) |
| II | 31 (52) | 19 (43) |
| III | 10 (17) | 10 (23) |
| Nodes | | |
| NK | 13 | 11 |
| O | 17 | 15 |
| 1–3 | 22 | 9 |
| 4+ | 7 | 9 |
| ER Status (%) | | |
| +ve | 65 | 62 |
| −ve | 35 | 38 |

IDC – infiltrating ductal carcinoma; ILC – infiltrating lobular carcinoma; NK – not known; ER – oestrogen receptor.

**Figure 1** Relation between Ki67 LI and histological grade. Bars represent median values. The increase in Ki67 LI between grades I and III, and grades II and III was statistically significant ($P<0.05$ by one-tailed Mann-Whitney U-test). The increase between grades I and II failed to reach significance ($P=0.12$).

**Figure 2** Relation between Ki67 LI and oestrogen receptor (ER) status. Bars represent median values. The difference was significant by the Mann-Whitney U-test ($P=$ 0.011).

*Effect of tamoxifen*

Tamoxifen or placebo was administered immediately after the first biopsy was taken and continued up until second biopsy at surgery, a median of 21 days later. The 59 patients who received tamoxifen had a median Ki67 LI of 5.6% in the first biopsy falling to 3.0% in the second biopsy (Figure 3; $P<0.001$ by Wilcoxon's matched-pairs signed-rank test). The 44 patients who received placebo had a median Ki67 LI of 5.4% in the first biopsy and 5.75% in the second (Figure 3), which was not significantly different by Wilcoxon's matched-pairs signed-rank test. No significant differences were seen when the median %Ki67 LI's of the first biopsy were compared between the two treatment groups, but there was a statistical difference when the post-treatment samples were compared ($P=0.002$ by Mann-Whitney U-test). The median change in %Ki67 LI between first and second

**Figure 3** Relation between Ki67 LI and treatment. Bars represent median values. In the tamoxifen-treated tumours, the Ki67 LI declined from a pre-treatment median level of 5.6% to a post-treatment median level of 3.0% ($P<0.001$ by Wilcoxon's matched-pairs signed-rank test). In the placebo-treated tumours there was no significant difference using the same test, the median Ki67 LI was 5.4% pre-treatment and 5.75% post-treatment.

tumour samples was −1.8% in the tamoxifen-treated group and +0.5% in those patients administered placebo; this difference in the change of %Ki67 LI was statistically significant by the Mann-Whitney U-test ($P=0.006$). In the tamoxifen-treated tumours there was no relationshp between a decline in %Ki67 LI and oestrogen receptor status: 69% of tumours exhibiting a greater than median change in %Ki67 LI (median change = −1.8%) were ER+ve, compared with 62% of tumours exhibiting a less than median change ($P>0.1$ by Chi-squared test). There was no correlation between the change in %Ki67 LI and the length of tamoxifen treatment (rho = −0.08 using Spearman's non-parametric correlation). Intra-tumoural heterogeneity was assessed by comparing the pre- and post-treatment Ki67 LI for the placebo group which showed them to be correlated using Spearman's non-parametric correlation coefficient (rho = 0.41, $P<0.007$).

In total, 44 out of 59 (74%) tamoxifen-treated tumours showed a decline in %Ki67 LI whereas only 19 out of 44 (43%) placebo-treated tumours had a negative change in %Ki67 LI ($P<0.001$ by Chi squared test). Tamoxifen had no significant effect on %ER or %PR staining assessed by Wilcoxon matched-paired signed-rank test. The median %ER was 10.5% (range 0–100, $n=54$) before tamoxifen treatment, and 21% (range 0–75, $n=54$) after treatment ($P=0.497$). In placebo group the pre-treatment median %ER was 0% (range 0–80, $n=41$) and 14.5% (range 0–82, $n=41$; $P=0.94$) after treatment. The median %PR was 9.5% (range 0–100, $n=54$) before, and 11% (range 0–100, $n=54$) after tamoxifen treatment ($P=0.233$), whilst in the placebo group the pre-treatment median %PR was 0% (range 0–100, $n=42$), and the post-treatment median %PR was 7.5% (range 0–75, $n=42$; $P=0.25$).

### *Patient outcome*

Disease-free survival was investigated over a short follow-up period (median = 18 months) and we found no difference in the rates of recurrence between patients whose tumours were either above or below the median %Ki67 LI in the first biopsy (data not shown). The change in %Ki67 LI (using the median change −1.8% as cut-off value) between the pre- and post-treatment biopsies in the tamoxifen treatment group also did not predict recurrence in our study.

### Discussion

We have attempted to further elucidate the relationship between Ki67 LI, ER and PR content, prognosis and the likelihood of response to tamoxifen therapy by looking at changes in these parameters over a short period of treatment (median 21 days). There was a pronounced change in the median Ki67 LI in the group receiving tamoxifen which might be expected, as tamoxifen is highly anti-proliferative for human breast tumour cells in culture. However, this is the first time that these anti-proliferative effects have been demonstrated using the Ki67 antibody on human tumours *in vivo*.

The staining of the tumours with the Ki67 antibody to the proliferating cell-associated antigen and the scoring of the percentage of stained nuclei yields a labelling index (LI) that represents the number of cells in the G1, S, G2 and M phases of the cell cycle. Other workers have shown that the Ki67 LI is positively correlated with histological grade (Gerdes *et al*., 1986; McGurrin *et al*., 1987; Lelle *et al*., 1987; Barnard *et al*., 1987; Walker & Camplejohn, 1988; Raymond & Leong, 1989; Wrba *et al*., 1989) and negatively correlated with oestrogen receptor content determined both immunohistologically and biochemically (Gerdes *et al*., 1987; Charpin *et al*., 1989; Bouzubar *et al*., 1989; Raymond & Leong, 1989; Wrba *et al*., 1989; Colley *et al*., 1989; Vollmer *et al*., 1989; Helin *et al*., 1989) and data obtained from our study are in agreement with these findings. Overall, the breast tumours in our study had a low mean Ki67 LI (7.0%) in comparison to other studies which report a range of mean Ki67 LI's from 7.2% to 22% (Wintzer *et al*., 1991). However, our value was similar to that of Wrba *et al*. (1989) (7.2%) whose method of scoring the tissue sections was similar to ours, i.e. 1,000 cells across the section. Some of the other groups whose mean values were somewhat higher scored the sections in a different manner, choosing to score, for instance, the areas of the section that were highly positive (Wintzer *et al.*, 1991). In other cases, there were less evident reasons for the disparity in mean Ki67 LI as the scoring criteria appeared similar to ours and it is assumed that there must be some methodological differences. The staining that was observed in our study appeared largely uniform throughout most tumour samples and this is confirmed by good correlation between the Trucut needle biopsies and the biopsies obtained at surgery in the placebo-treated group.

We were unable to show induction of PR with tamoxifen treatment. It is possible that this is related to the period of time for which our patients were treated (median of 21 days) which is considerably longer than the 8 days used in our previous study (Howell *et al.*, 1987*a*) or the 3 and 7 days used by Noguchi *et al.* (1988). Noguchi *et al.* (1988) further showed that continuation of tamoxifen treatment to 14 days abolished stimulation of PR synthesis presumably because its initial agonist effects are soon reversed by its antagonism of oestrogen action. The absence of PR induction may also be related to the use of the immunocytochemical method of PR measurement which is only semiquantitative and ignores the intensity of staining.

Further analysis of the tamoxifen-treated group showed no relationship between a change in Ki67 LI and tumour steroid receptor content. One might expect the 'responsive' (↓ Ki67 LI, $n = 44$) group to contain more receptor-positive (particularly PR) tumours than the 'unresponsive' (↑ Ki67 LI, $n = 15$) patients. However, receptor status was evenly distributed between the two groups and the medians of the receptor levels were also not significantly different. This lack of correlation may be a true reflection of the *in vivo* state during short-term tamoxifen treatment, but conflicts with the reported 75% response rate in ER positive PR positive tumours compared with a 10% response rate in ER negative PR negative tumours (McGuire & Clark, 1983). The disparity may be due to heterogeneity in receptor status within each carcinoma (Osborne, 1985; Alanko, 1985; Van Netten *et al.*, 1986; Howell *et al.*, 1987*b*), and between the Trucut and surgical biopsies (Jackesz *et al.*, 1985; Young *et al.*, 1985). This could well have influenced our study as receptor staining and Ki67 staining were not performed on adjacent histological sections as receptors are stained routinely in the laboratory when breast tumour specimens arrive, whereas the Ki67 antibody staining was performed at a later date. It may well be that Ki67 staining (i.e. proliferation) varies throughout a tumour in a similar fashion to steroid receptor levels and that if staining were performed on adjacent sections a correlation could perhaps be seen.

We also investigated disease-free survival over a short follow-up period (median = 18 months). We found no difference in the rates of recurrence between patients whose tumours were either above or below the median Ki67 LI in the first biopsy. The change in Ki67 LI (using the median change as cut-off value) between the pre- and post-treatment biopsies in the tamoxifen treatment group also did not predict recurrence in our study. However, longer follow-up may reveal Ki67 LI to be a prognostic factor as shown in the study by Wintzer *et al.* where patients were followed up for a median of 37 months. Using the median as cut-off point, they found significantly different curves for disease-free survival, but Ki67 LI was of independent prognostic value only if a higher cut-off level was selected.

To our knowledge, this is the first study in which short-term tamoxifen treatment of breast tumour *in vivo* has been shown to lead to a significant fall in proliferation as measured by Ki67 immunostaining. This change in proliferative status was not correlated with other indicators of hormone responsiveness such as receptor status. There was no relationship between Ki67 LI and recurrence rates of all patients or between changes in Ki67 LI from first to second biopsy and recurrence rates in the tamoxifen treated group, although the follow-up period is short. The reduction in Ki67 immunostaining in tamoxifen-treated patients suggests that the antibody Ki67 may prove to be of value in determining response for both established therapies such as tamoxifen treatment and trial therapies, for example when studying the efficacy of the new, pure antioestrogens.

We are most grateful to ICI pharmaceuticals plc for providing the 'Nolvadex' (tamoxifen) and placebo tablets for this study. This work is supported by the Endowment Fund of the Christie Hospital NHS Trust.

### References

ALANKO, A. (1985). Variation of estrogen and progesterone receptor status in breast cancer. *Ann. Clin. Res.*, **17**, 10-14.

BARNARD, N.J., HALL, P.A., LEMOINE, N.R. & KADAR, N. (1987). Proliferative index in breast carcinoma determined *in situ* by Ki67 immunostaining and its relationship to clinical and pathological variables. *J. Pathol.*, **152**, 287-295.

BOUZUBAR, N., WALKER, K.J., GRIFFITHS, K., ELLIS, I.O., ELTSON, C.W., ROBERTSON, J.F.R., BLAMEY, R.W. & NICHOLSON, R.I. (1989). Ki67 immunostaining in primary breast cancer: pathological and clinical associations. *Br. J. Cancer*, **59**, 943-947.

CHARPIN, C., ANDRAC, L., HABIB, M.-C., VACHERET, H., XERRI, L., DEVICTOR, B., LAVAUT, M.N. & TOGA, M. (1989). Immunodetection in fine needle aspirates and multiparametric (SAMBA) image analysis: receptors (monoclonal antiestrogen and antiprogesterone) and growth fraction (monoclonal Ki67) evaluation in breast carcinomas. *Cancer*, **63**, 863-872.

COLLEY, M., KOMMOS, F., BIBBO, M., DYTCH, H.E., FRANKLIN, W.A., HOLT, J.A. & WIED, G.L. (1989). Assessment of hormone receptors in breast carcinomas by immunocytochemistry and image analysis. *Anal. Quant. Cytol. Histol.*, **11**, 307-314.

GERDES, J., LEELE, R.J., PICKARTZ, H., HEIDENREICH, W., SCHWARTING, R., KURTSIEFER, L., STAUCH, G. & STEIN, H. (1986). Growth fractions in breast cancers determined *in situ* with monoclonal antibodies Ki67. *J. Clin. Pathol.*, **39**, 977-980.

GERDES, J., LEMKE, H., BAISCH, H., WACKER, H.-H., SCHWAB, U. & STEIN, H. (1984). Cell cycle analysis of a proliferation-associated human nuclear antigen defined by the monoclonal antibody Ki67. *J. Immunol.*, **133**, 1710-1715.

GERDES, J., PICKHARTZ, H., BROTHERTON, J., HAMMERSTEIN, J., WEITZEL, H. & STEIN, H. (1987). Growth fractions and estrogen receptors in human breast cancers as determined *in situ* with monoclonal antibodies. *Am. J. Pathol.*, **129**, 486-492.

GERDES, J., SCHWAB, U., LEMKE, H. & STEIN, H. (1983). Production of a mouse monoclonal antibody reactive with a human nuclear antigen associated with cell proliferation. *Int. J. Cancer*, **31**, 13-20.

HELIN, M.L., HELLE, M.J., HELIN, H.J. & ISOLA, J.J. (1989). Proliferative activity and steroid receptors determined by immunohistochemistry in adjacent frozen sections of 102 breast carcinomas. *Arch. Pathol. Lab. Med.*, **113**, 854-857.

HORWITZ, K.B., YOSEKI, Y. & MCGUIRE, W.L. (1978). Estrogen control of progesterone receptor in human breast cancer: role of estradiol and antiestrogen. *Endocrinology*, **103**, 1742-1751.

HOWELL, A., HARLAND, R.N.L., BARNES, D.M., BAILSDAM, A.D., WILKINSON, M.J.S., HAYWARD, E., SWINDELL, R. & SELLWOOD, R.A. (1987*a*). Endocrine therapy for advanced carcinoma of the breast: relationship between the effect of tamoxifen upon concentrations of progesterone receptor and subsequent response to treatment. *Cancer Research*, **47**, 300-304.

HOWELL, A., HARLAND, R.N.L., BARNES, D.M., HAYWARD, E., REDFORD, J., SWINDELL, R. & SELLWOOD, R.A. (1987*a*). Endocrine therapy for advanced carcinoma of the breast: effect of tumour heterogeneity and site of biopsy on the predictive value of progesterone receptor estimations. *Cancer Res.*, **47**, 296-299.

JAKESZ, R., DITTRICH, C., HANUSCH, J., KOLB, R., LENZHOFER, R., MOSER, K., RAINER, H., REINER, G., SCHEMPER, M. & SPONA, J. (1985). Simultaneous and sequential determinations of steroid hormone receptors in human breast cancer. Influence of intervening therapy. *Ann. Surg.*, **201**, 305-310.

LELLE, R.J., HEINDENREICH, W., STAUCH, G. & GERDES, J. (1987). The correlation of growth fractions with histologic grading and lymph node status in human mammary carcinoma. *Cancer*, **56**, 83-88.

MCGUIRE, W.L. & CLARK, G.M. (1983). The prognostic role of progesterone receptors in human breast cancer. *Semin. Oncol.*, **10**, 2-6.

MCGURRIN, J.F., DORIA, M.I., DAWSON, P.J., KARRISON, T., STEIN, H.O. & FRANKLIN, W.A. (1987). Assessment of tumour cell kinetics by immunohistochemistry in carcinoma of the breast. *Cancer*, **59**, 1744-1750.

MEYER, J.S., PREY, M.V., BABCOCK, D.S. & MCDIVITT, R.W. (1986). Breast carcinoma cell kinetics, morphology, stage and host characteristics. *Lab. Invest.*, **54**, 41-51.

MEYER, J.S., RAO, B.R., STEVENS, S.C. & WHITE, W.L. (1977). Low incidence of estrogen receptor in breast carcinomas with rapid rates of cellular proliferation. *Cancer*, **40**, 2290-2298.

NOGUCHI, S., MIYAUCHI, K., NISHIZAWA, Y. & KOYAMA, H. (1988). Induction of progesterone receptor with tamoxifen in human breast cancer with special reference to its behaviour over time. *Cancer*, **61**, 1345-1349.

OLSZEWSKI, W., DARZYNKIEWICZ, Z., ROSEN, P.P., SCHARTZ, M.K. & MELAMED, M.R. (1981). Flow cytometry of breast carcinoma, II: relation of tumour cell cycle distribution to histology and estrogen receptor. *Cancer*, **48**, 985-988.

OSBORNE, C.K. (1985). Heterogeneity in hormone receptor status in primary and metastatic breast cancer. *Semin. Oncol.*, **12**, 317-326.

RABER, M.N., BARLOGIE, B., LATREILLE, J., BEDROSIAN, C., FRITSCHE, H. & BLUMENSCHEIN, G. (1982). Ploidy, proliferative activity and estrogen receptor content in human breast cancer. *Cytology*, **3**, 36-41.

RAYMOND, W.A. & LEONG, A.S.Y. (1989). The relationship between growth fractions and oestrogen receptors in human breast carcinoma, as determined by immunohistochemical staining. *J. Pathol.*, **8**, 203-211.

SUNDERLAND, M.C. & MCGUIRE, W.L. (1991). Hormones and breast cancer. *Trends Endocrinol. Metab.*, **2**, 72-76.

VAN NETTEN, J.P., COY, P., BRIGDEN, M.L., GALLAGHER, S., CARLISLE, S.J. & THORNTON, I. (1986). Intermediate estrogen receptor levels in human breast cancer. *Eur. J. Cancer Clin. Oncol.*, **22**, 1543-1545.

VOLLMER, G., GERDES, J. & KNUPPEN, R. (1989). Relationship of cytosolic estrogen and progesterone receptor content and the growth fraction in human mammary carcinomas. *Cancer Res.*, **49**, 4011-4014.

WALKER, R.A. & CAMPLEJOHN, R.S. (1988). Comparison of monoclonal antibody Ki67 reactivity with grade and DNA flow cytometry of breast carcinomas. *Br. J. Cancer*, **57**, 281-283.

WINTZER, H.-O., ZIPFEL, I.,. SCHULTE-MONTING, J., HELLERICH, U. & VON KLEIST, S. (1991). Ki67 immunostaining in human breast tumours and its relationship to prognosis. *Cancer*, **67**, 421-428.

WRBA, F., CHOTT, A., REINER, A., REINER, G., MARKIS-RITZINGER, E. & HOLZNER, J.H. (1989). Ki67 immunoreactivity in breast carcinomas in relation to transferrin receptor expression, estrogen receptor status and morphological criteria. *Oncology*, **46**, 255-259.

YOUNG, S.C., BURKETT, R.J. & STEWART, C. (1985). Discrepancy in ER levels of breast carcinoma in biopsy vs mastectomy specimens. *J. Surg. Oncol.*, **29**, 54-56.